# Exercise-Induced Bone Formation Is Poorly Linked to Local Strain Magnitude in the Sheep Tibia

**DOI:** 10.1371/journal.pone.0099108

**Published:** 2014-06-04

**Authors:** Ian J. Wallace, Brigitte Demes, Carrie Mongle, Osbjorn M. Pearson, John D. Polk, Daniel E. Lieberman

**Affiliations:** 1 Department of Anthropology, Stony Brook University, Stony Brook, New York, United States of America; 2 Department of Anatomical Sciences, Stony Brook University, Stony Brook, New York, United States of America; 3 Department of Anthropology, University of New Mexico, Albuquerque, New Mexico, United States of America; 4 Department of Anthropology, University of Illinois at Urbana-Champaign, Urbana, Illinois, United States of America; 5 Department of Human Evolutionary Biology, Harvard University, Cambridge, Massachusetts, United States of America; University of Utah, United States of America

## Abstract

Functional interpretations of limb bone structure frequently assume that diaphyses adjust their shape by adding bone primarily across the plane in which they are habitually loaded in order to minimize loading-induced strains. Here, to test this hypothesis, we characterize the *in vivo* strain environment of the sheep tibial midshaft during treadmill exercise and examine whether this activity promotes bone formation disproportionately in the direction of loading in diaphyseal regions that experience the highest strains. It is shown that during treadmill exercise, sheep tibiae were bent in an anteroposterior direction, generating maximal tensile and compressive strains on the anterior and posterior shaft surfaces, respectively. Exercise led to significantly increased periosteal bone formation; however, rather than being biased toward areas of maximal strains across the anteroposterior axis, exercise-related osteogenesis occurred primarily around the medial half of the shaft circumference, in both high and low strain regions. Overall, the results of this study demonstrate that loading-induced bone growth is not closely linked to local strain magnitude in every instance. Therefore, caution is necessary when bone shaft shape is used to infer functional loading history in the absence of *in vivo* data on how bones are loaded and how they actually respond to loading.

## Introduction

Functional analyses of limb bone structure, without prior knowledge of loading history, commonly assume that the distribution of bony material within diaphyseal cross sections is affected by the magnitude of local mechanical strains (deformations) generated by habitual physical activities such as locomotion [1]. In this strategy, bone tissue's formative and resorptive activity is considered an optimization process that acts primarily to maximize resistance to loading-induced strains with a minimum of material. Functional loading is expected to have a predictable effect on diaphyseal shape by promoting bone formation disproportionately in areas of the shaft surface that are routinely subject to the highest levels of strain. Thus, bone shaft cross-sectional shapes that display structural reinforcement in a particular plane (e.g., elliptically shaped cross sections) are hypothesized to have experienced more frequent bending loads and higher strains across the axis about which the bulk of bone tissue is distributed [2]–[4].

However, studies documenting *in vivo* diaphyseal strains in a variety of animals have shown that limb bone shaft cross sections are not always reinforced in the planes in which they are habitually loaded [5]–[8]. For example, during quadrupedal walking, the ulna of a macaque monkey primarily experiences coronal bending, with the highest strains produced on the medial and lateral shaft surfaces; however, macaque ulnar diaphyses are shaped to better resist sagittal bending [7]. Some functional morphologists have downplayed the relevance of these results by pointing to other strain studies that found the correspondence between maximum strain distribution and maximum bending rigidity in diaphyses to improve during more vigorous activities [1]. For example, when macaques transition from walking to galloping, the greatest strains engendered in tibial shafts begin to approximate the direction of maximal diaphyseal bending rigidity [9]. This suggests to some that diaphyseal shape indeed reflects patterns of functional loading, particularly activities involving vigorous loading [1].

In order to rigorously evaluate the relationship between limb bone loading history and diaphyseal shape, experiments are required that directly relate *in vivo* functional strains to patterns of bone growth. To date, however, few studies of this kind have been conducted. In the present study, we characterize the *in vivo* strain environment of the tibial midshaft in sheep during treadmill exercise and test whether bone formation induced by this activity occurs primarily in diaphyseal regions that experience the highest strains. Such a growth response would structurally reinforce the shaft across its bending axis and, likewise, would influence diaphyseal shape in a manner consistent with the model within which functional morphologists often interpret shaft structure in the absence of information on loading history.

## Materials and Methods

### Ethics Statement

All experimental procedures were reviewed and approved by the Institutional Animal Care and Use Committee of Harvard University.

### Experimental Procedures

Juvenile animals were employed because the osteogenic response to exercise is typically greatest during the growth period [10]–[12]. At 40 days of age, rams (*Ovis aries*; Dorset) were divided into three groups. In the first group (N = 5), strain gauges were attached to the tibial midshaft, and strain data were recorded during treadmill trotting at a Froude speed of 0.5 (approximately 4 km h^−1^). A second group (N = 5) was exercised on a treadmill at the same Froude speed for 30 min day^−1^ (approximately 6,000 loading cycles day^−1^) for 90 days. Subjects were injected (i.p.) with calcein (20 mg kg^−1^) after the first week of the exercise treatment to record bone growth on the periosteal surface of the tibia. The third group (N = 5) served as sedentary controls and received a calcein injection but no exercise treatment. All subjects were individually housed and were given the same *ad libitum* diet of food and water. After the 90-day treatment period, subjects were euthanized and tibiae were extracted.

### Strain Gauge Recordings

Following induction and maintenance of isoflurane general anaesthesia, triple-rosette strain gauges (Sokki Kenkyujo, Tokyo, Japan) were affixed to the anterior, medial, and posterior diaphyseal surfaces of the left tibia through small skin incisions using aseptic technique. At each gauge site, 5 mm^2^ of periosteum was removed, the bone surface was degreased with 100% chloroform, and a gauge was glued to the prepared surface with methyl-2-cyano-acrylate. Care was taken to align one gauge element with the long axis of the bone. Gauge leads were passed extracutaneously beneath flexible bandages to the hip, where they were sutured to a belt worn around the subject's abdomen. Strain relief was provided by attaching the leads to dressing wrapped around the leg near the incision sites.

Strain data were recorded 4 and 24 h after surgery, when subjects were moving with normal gaits and showed no signs of lameness or discomfort. During each recording session, gauges were connected with insulated wire to Vishay 2120A amplifiers (MicroMeasurements Inc., Raleigh, NC) to form one arm of a Wheatstone quarter-bridge circuit. Bridge excitation was 1 V. Voltage outputs were recorded on a Teac RD-145T DAT tape recorder (Teac Corp., Tokyo, Japan). Gauges were calibrated when subjects were stationary with their instrumented limb off the ground, and were periodically balanced during experiments to adjust for zero offsets.

### Strain Gauge Analyses

Strain data sequences were sampled from tape recordings using an Ionet A-D board (GW Instruments, Somerville, MA) at 250 Hz. A custom-written Superscope 3.0 (GW Instruments, Somerville, MA) virtual instrument was used to determine the zero offset, calculate strains of principal tension and compression from raw voltage data using shunt calibration signals recorded during experiments, and calculate the orientation of principal tensile strain relative to the bone's long axis using standard engineering equations [13]. Igor Pro 4.0 (Wavemetrics Inc., Lake Oswego, OR) was used to calculate these strains at temporal midstance—when peak strains occur [8]—for at least 10 gait cycles per subject. To characterize the average tibial midshaft strain environment among all subjects, a custom-written macro [8] for ImageJ (NIH, Bethesda, MD) was used to compute the mean location and orientation of the neutral axis, as well as mean gradients of cross-sectional normal strain, using linear beam theory [13].

### Histological Analyses

**Figure 1:**
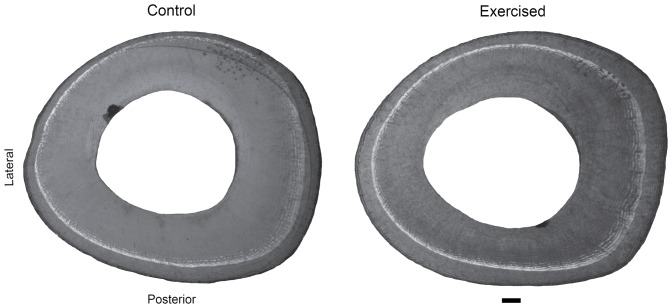
Tibial midshaft cross sections. The sedentary control sheep (subject 4) and the exercised sheep (subject 3) had similar body masses throughout the experiment. The calcein labels injected after the first week of the exercise treatment are visible as the light grey rings within the sections. Scale equals 1 mm.

In the exercised and control subjects, bone formation on the tibial periosteal surface during the treatment period was quantified on midshaft transverse cross sections (100-µm thickness) using an Olympus SZH-10 microscope (Olympus America, Melville, NY) with epifluorescence (Fig. 1). Using ImageJ, digitized cross sections were divided into 16 equal-angle sectors positioned about the experimentally determined neutral axis, with the axis orthogonal to the neutral axis projected through the area centroid [8]. Bone area added in each sector during the treatment period was measured from the calcein line to the periosteal surface. Bone areas were standardized by body mass^0.67^, calculated as mean mass during the final 3 weeks of the experiment. Two-sample Wilcoxon tests were performed to evaluate differences in bone formation between exercised and control animals.

Additional details of the experimental and analytical methods are provided elsewhere [8], [11]. Raw bone strain and histological data are available in [8] and Dataset S1, respectively.

## Results and Discussion

**Figure 2:**
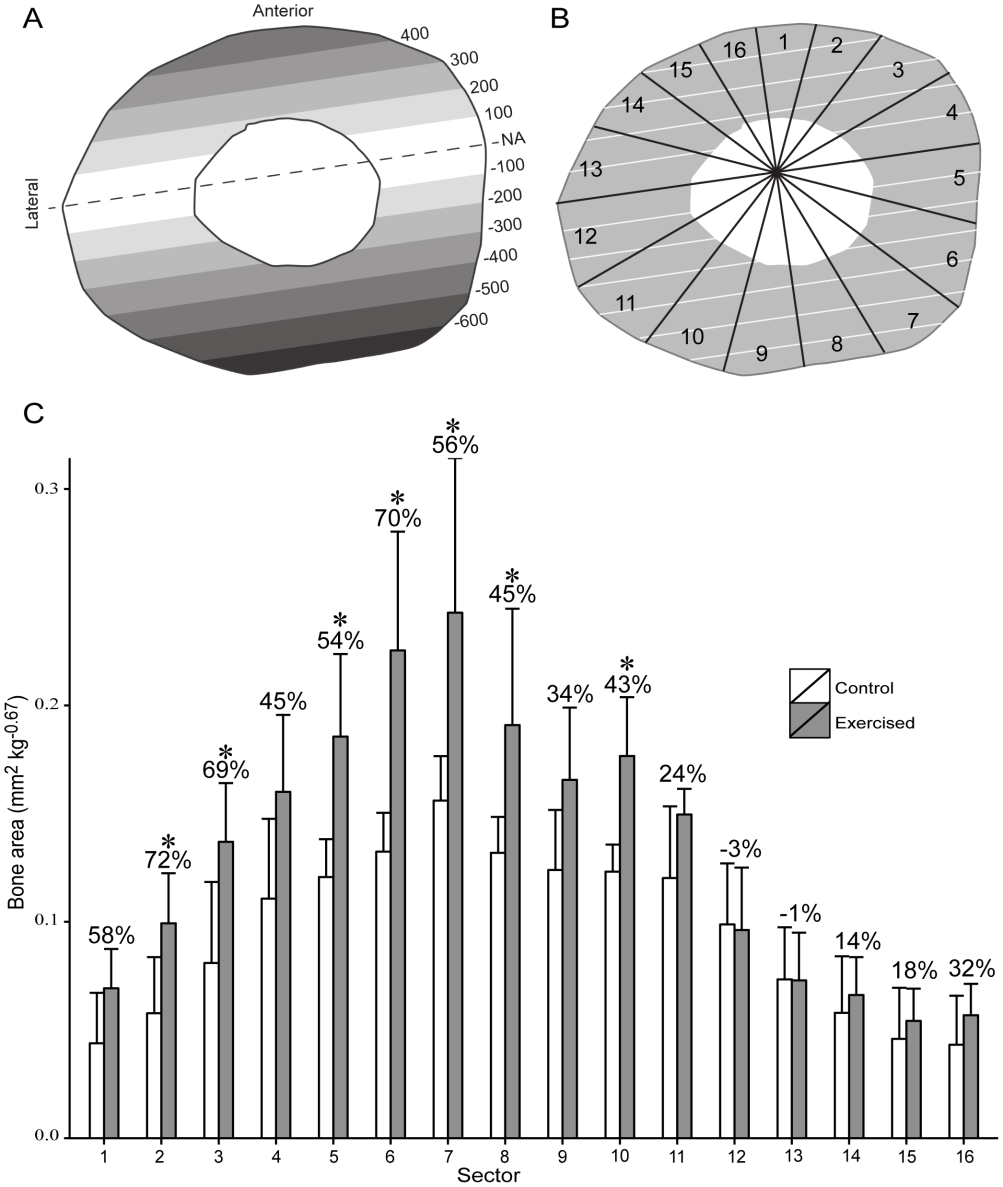
Bone strain and bone formation. (A) Mean distribution of peak longitudinal normal strain (µε) across the tibial mid-diaphysis during treadmill exercise. The neutral axis (NA) is dashed with compressive and tensile strain isopleths plotted parallel to it. Maximal compressive strains (negative sign) were produced on the posterior shaft surface, and maximal tensile strains were produced on the anterior shaft surface. (B) Tibial midshaft cross section subdivided into 16 equal-angle sectors positioned about the neutral axis, with the axis orthogonal to the neutral axis projected through the area centroid. (C) Distribution of periosteal bone added in exercised animals and sedentary controls during the experimental period (means + SD). Asterisks indicate statistically significant (*P*<0.05) differences between exercised animals and controls as determined by two-sample Wilcoxon tests. Numbers indicate percent difference between the mean of the exercised animals relative to the control mean.

Treadmill exercise caused a highly non-uniform strain environment in the sheep tibial midshaft (Fig. 2A). During peak loading, maximal compressive strains were generated on the posterior shaft surface, and maximal tensile strains were produced on the anterior surface. The neutral axis was oriented within 10° of a mediolateral axis and shifted toward the anterior diaphyseal surface, indicating that bones were bent in an anteroposterior direction. Maximal compressive strains were approximately 40% higher than tensile strains, as expected in a loading regime that includes both bending and axial compression. Based on these data, if functional loading stimulates bone formation disproportionately in diaphyseal regions that experience the highest strains, as is often assumed in functional interpretations of bone shaft structure, then one would expect treadmill exercise to promote osteogenesis primarily on the anterior and posterior tibial shaft surfaces.

However, in the sheep in this study, locations of exercise-induced bone formation were poorly linked to local strain magnitude during peak loading (Fig. 2B,C). Instead of being biased toward areas of maximal strains across the anteroposterior shaft axis, significant exercise-induced bone formation occurred primarily around the medial half of the diaphyseal circumference, in both high strain regions and directly adjacent to the neutral axis where strains were lowest. In nearly all areas of the lateral shaft surface, exercise failed to stimulate significant bone formation, including in multiple sectors that experienced maximal strains. Relative differences in bone added between exercised sheep and sedentary controls were greatest in an area of high tensile strains (sector 2) and smallest in the two lateral sectors adjacent to the neutral axis (sectors 12 and 13), yet relative group differences were very similar between other regions of maximal and minimal strains (e.g., sector 4 vs. 8). In sum, these results demonstrate that, although it might be assumed that the primary purpose of bone response to loading is to adjust its morphology to best resist maximal strains, this is not necessarily what is achieved in every instance.

These findings accord well with those of previous experiments with bird models involving both exogenous limb loading [14] and weight-bearing exercise [6] that failed to find any significant correlation between bone formation stimulated by loading and local strain magnitude. In contrast, however, peak magnitude strains have been associated with sites of increased bone formation in experiments involving external loading of rodent limbs [15], [16] and natural physiological limb loading in goats [17]. A notable difference between these previous studies is that in the bird experiments, subjects were skeletally mature and the surfaces of their bones were primarily quiescent, whereas subjects in the mammal experiments were growing and had bones with active surfaces. Bone's response to loading is known to be affected by age [10]–[12], and has been hypothesized to differ between distantly related taxa [1], [18]. Therefore, the agreement between the results of bird studies and the present experiment involving growing mammals is important because it implies that the bone functional response observed in sheep was not due to their specific age, nor is it unusual for vertebrates in general. Also important is the disparity between our results and those of other mammal studies because it suggests that a universal relationship between bone growth and local strain magnitude probably does not exist.

Previous *in vivo* strain studies demonstrating that, in many animals, limb bone shafts are structurally reinforced about the bending axis rather than in the direction of bending have led to the suggestion that bone turnover may not be optimized to minimize strains, but instead to confine strains to a relatively narrow range of orientations [5], [6]. According to this view, loading-induced bone formation should occur primarily in low strain areas across the neutral axis, rather than in high strain regions, as a way of ensuring that bones are loaded in a predictable fashion. In the present study, however, although significant bone formation was stimulated by exercise near the neutral axis (at least on the medial shaft surface), it was also induced in areas distant from the neutral axis in the plane of maximum strain distribution. Thus, our results are inconsistent with hypotheses that limb bone shaft shape results from an adaptive process that either minimizes strains or makes them more predictable. Nevertheless, it possible that the patterns of exercise-induced bone formation observed in this study reflect some sort of compromise between maintaining equilibrium strain levels while still ensuring that bones maintain a sufficient safety factor to withstand atypical loading [17].

The lack of a predictable relationship between bone formation and strain magnitude highlights the inherent problem with inferring loading history from bone shaft shape in the absence of *in vivo* data on how bones are loaded and how they actually respond to loading. Without a doubt, functional loading has a critical influence on limb bone shaft morphology, and strains either directly or indirectly (e.g., through cortical interstitial fluid flow) play an important role in regulating bone mechanoresponsiveness. However, strain parameters such as frequency [19], rate [20], orientation [21], and gradients [6], [14], may be as influential as magnitude in the control of bone turnover. Biophysical signals independent of strain, such as cellular oscillations, might also have an effect on bone growth [22]. Ultimately, the mechanical environment of limb bone shafts produced by functional loading is complex, and it is unlikely that any single dimension of loading history is solely responsible for causing changes in bone cell activity [23]. More likely, the distribution of bone tissue within diaphyses is affected by numerous aspects of the constant barrage of biophysical signals, spanning a wide range of magnitudes, to which bone cells are exposed throughout the daily course of functional activities. However, in terms of the precise local mechanical conditions under which bone turnover results in net formation (or resorption), gaps exist in our understanding. Until those gaps are filled, there is risk in assuming any simple relationship between limb bone diaphyseal shape and functional loading history [10].

## Supporting Information

Dataset S1 Contains raw body mass and histological data.
(XLS)

## References

[1] Ruff CB, Holt B, Trinkaus E (2006) Who's afraid of the big bad Wolff? “Wolff's Law” and bone functional adaptation. Am J Phys Anthropol 129: 484–498. 16425178 10.1002/ajpa.20371

[2] Larsen CS (1997) Bioarchaeology: Interpreting behavior from the human skeleton. Cambridge: Cambridge University Press. 473 p.

[3] Ruff CB (2008) Biomechanical analyses of archaeological human skeletons. In: Katzenberg MA, Saunders SR, editors. Biological anthropology of the human skeleton.New York: Alan R. Liss. pp. 183–206.

[4] Moore MK (2013) Functional morphology and medical imaging. In: DiGangi EA, Moore MK, editors. Research methods in human skeletal biology.Waltham, MA: Academic Press. pp. 397–424.

[5] Lanyon LE, Rubin CT (1985) Functional adaptation in skeletal structures. In: Hildebrand M, Bramble DM, Liem KF, Wake BD, editors. Functional vertebrate morphology.Cambridge, MA: Belknap Press. pp. 1–25.

[6] Judex S, Gross TS, Zernicke RF (1997) Strain gradients correlate with sites of exercise-induced bone-forming surfaces in the adult skeleton. J Bone Miner Res 12: 1737–1745. 9333136 10.1359/jbmr.1997.12.10.1737

[7] Demes B, Stern JT Jr, Hausman MR, Larson SG, McLeod KJ, et al (1998) Patterns of strain in the macaque ulna during functional activity. Am J Phys Anthropol 106: 87–100. 9590526 10.1002/(SICI)1096-8644(199805)106:1<87::AID-AJPA6>3.0.CO;2-A

[8] Lieberman DE, Polk JD, Demes B (2004) Predicting long bone loading from cross-sectional geometry. Am J Phys Anthropol 123: 156–171. 14730649 10.1002/ajpa.10316

[9] Demes B, Qin Y-X, Stern JT Jr, Larson SG, Rubin CT (2001) Patterns of strain in the macaque tibia during functional activity. Am J Phys Anthropol 116: 257–265. 11745077 10.1002/ajpa.1122

[10] Bertram JEA, Swartz SM (1991) The “law of bone transformation”: a case of crying Wolff? Biol Rev 66: 245–273. 1932466 10.1111/j.1469-185x.1991.tb01142.x

[11] Lieberman DE, Pearson OM, Polk JD, Demes B, Crompton AW (2003) Optimization of bone growth and remodeling in response to loading in tapered mammalian limbs. J Exp Biol 206: 3125–3138. 12909694 10.1242/jeb.00514

[12] Pearson OM, Lieberman DE (2004) The aging of Wolff's “Law”: ontogeny and responses to mechanical loading in cortical bone. Yrbk Phys Anthropol 47: 63–99. 10.1002/ajpa.20155 15605390

[13] Biewener AA (1992) *In vivo* measurement of bone strain and tendon force. In: Biewener AA, editor. Biomechanics—structures and systems: a practical approach,Oxford: Oxford University Press. pp. 123–147.

[14] Gross TS, Edwards JL, McLeod KJ, Rubin CT (1997) Strain gradients correlate with sites of periosteal bone formation. J Bone Miner Res 12: 982–988. 9169359 10.1359/jbmr.1997.12.6.982

[15] Mosley JR, March BM, Lynch J, Lanyon LE (1997) Strain magnitude related changes in whole bone architecture in growing rats. Bone 20: 191–198. 9071468 10.1016/s8756-3282(96)00385-7

[16] Warden SJ, Fuchs RK, Turner CH (2004) Steps for targeting exercise towards the skeleton to increase bone strength. Eur Med Phys 40: 223–232. 16172590

[17] Main RP (2007) Ontogenetic relationships between *in vivo* strain environment, bone histomorphometry and growth in the goat radius. J Anat 210: 272–293. 17331177 10.1111/j.1469-7580.2007.00696.x PMC2100276

[18] Carter DR, Beaupré GS (2001) Skeletal function and form: Mechanobiology of skeletal development, aging, and regeneration. Cambridge: Cambridge University Press. 332 p.

[19] Rubin C, Turner AS, Bain S, Mallinckrodt C, McLeod K (2001) Anabolism: low mechanical signals strengthen long bones. Nature 412: 603–604. 10.1038/35088122 11493908

[20] O'Connor JA, Lanyon LE, MacFie H (1982) The influence of strain rate on adaptive bone remodelling. J Biomech 15: 767–781. 7153230 10.1016/0021-9290(82)90092-6

[21] Wallace IJ, Kwaczala AT, Judex S, Demes B, Carlson KJ (2013) Physical activity engendering loads from diverse directions augments the growing skeleton. J Musculoskelet Neuronal Interact 13: 283–238. 23989249

[22] Garman R, Rubin C, Judex S (2007) Small oscillatory accelerations, independent of matrix deformations, increase osteoblast activity and enhance bone morphology. PLoS ONE 2: e653. 17653280 10.1371/journal.pone.0000653 PMC1919432

[23] Thompson WR, Rubin CT, Rubin J (2012) Mechanical regulation of signaling pathways in bone. Gene 503: 179–193. 22575727 10.1016/j.gene.2012.04.076 PMC3371109

